# Hydrogen Sulfide Plays an Important Role by Regulating Endoplasmic Reticulum Stress in Diabetes-Related Diseases

**DOI:** 10.3390/ijms23137170

**Published:** 2022-06-28

**Authors:** Huijie Zhao, Huiyang Liu, Yihan Yang, Tianyue Lan, Honggang Wang, Dongdong Wu

**Affiliations:** 1 Institute of Chronic Disease Risks Assessment, Henan University, Jinming Avenue, Kaifeng 475004, China; zhj5696@163.com; 2 School of Basic Medical Sciences, Henan University, Kaifeng 475004, China; m15736875597@163.com (H.L.); h1323240458@163.com (Y.Y.); 3 School of Nursing and Health, Henan University, Jinming Avenue, Kaifeng 475004, China; 15137215980@163.com; 4 School of Stomatology, Henan University, Kaifeng 475004, China

**Keywords:** hydrogen sulfide, endoplasmic reticulum stress, diabetes-associated cognitive dysfunction, diabetes, diabetic cardiomyopathy

## Abstract

Endoplasmic reticulum (ER) plays important roles in protein synthesis, protein folding and modification, lipid biosynthesis, calcium storage, and detoxification. ER homeostasis is destroyed by physiological and pharmacological stressors, resulting in the accumulation of misfolded proteins, which causes ER stress. More and more studies have shown that ER stress contributes to the pathogenesis of many diseases, such as diabetes, inflammation, neurodegenerative diseases, cancer, and autoimmune diseases. As a toxic gas, H_2_S has, in recent years, been considered the third most important gas signal molecule after NO and CO. H_2_S has been found to have many important physiological functions and to play an important role in many pathological and physiological processes. Recent evidence shows that H_2_S improves the body’s defenses to many diseases, including diabetes, by regulating ER stress, but its mechanism has not yet been fully understood. We therefore reviewed recent studies of the role of H_2_S in improving diabetes-related diseases by regulating ER stress and carefully analyzed its mechanism in order to provide a theoretical reference for future research.

## 1. Introduction

Endoplasmic reticulum (ER) is an important organelle in eukaryotic cells, which is responsible for the synthesis and modification of lipids, proteins, and carbohydrates, as well as the regulation of intracellular calcium concentration [1,2]. When a variety of physiological and pathological factors destroy the steady-state balance of ER, ER stress is triggered, producing a large number of unfolded or misfolded proteins, and leading to disorder in calcium concentration and lipid synthesis. The accumulation of the unfolded and misfolded proteins in ER induces ER stress and unfolded protein response (UPR) [3]. Moderate ER stress can restore ER homeostasis and make cells adapt to environmental changes. Intense and sustained ER stress, however, can induce caspase-12-dependent apoptosis, resulting in a variety of diseases [4].

Hydrogen sulfide (H_2_S) is a flammable, volatile, and toxic gas with the smell of rotten eggs [5]. Evidence indicates that H_2_S has a variety of biological functions [6] and plays a role in many diseases [3], including diabetes-related diseases [7]. The literature has shown that H_2_S regulates ER stress in a variety of pathological processes, including sepsis-induced myocardial dysfunction [8], diabetes [9], hepatic ischemia/reperfusion injury [10], and cardiomyopathy [11]. However, the exact mechanism has not yet been fully understood, especially as to the role of H_2_S in regulating ER stress in diabetes. In this review, we considered recent literature on the important role of H_2_S in regulating ER stress in diabetes and analyzed its mechanism, hoping to provide a theoretical reference for exploring the role of H_2_S in improving future diabetes treatments by regulating ER stress.

## 2. Overview of Endoplasmic Reticulum Stress

**Figure 1:**
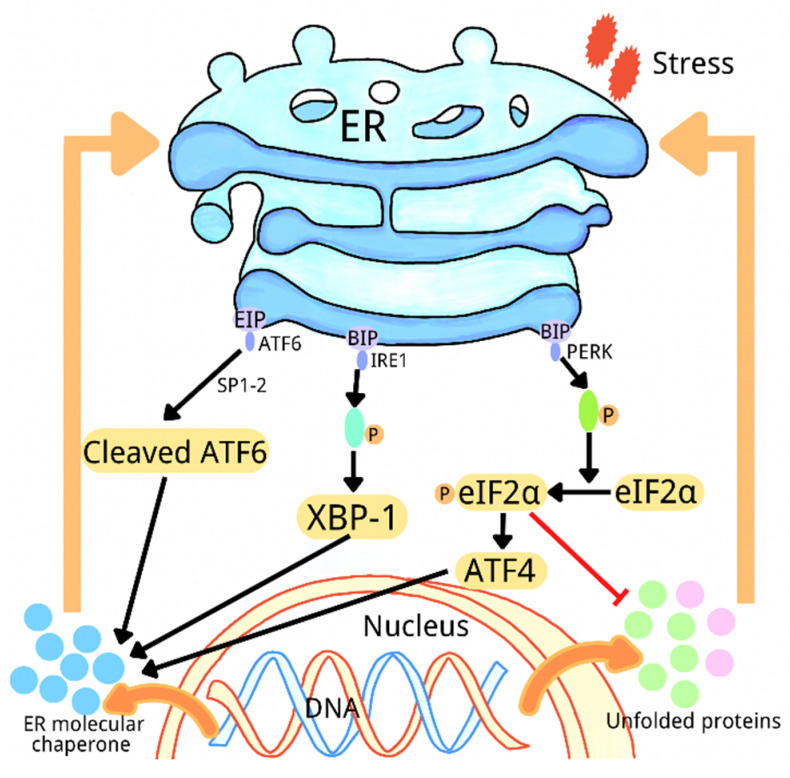
A schematic diagram of three signal pathways of endoplasmic reticulum stress and UPR.

The ER is an important organelle for protein synthesis, folding, and modification and for calcium storage and lipid synthesis [12]. Many physiological and pathological factors, including inflammation, hypoxia, glucose deficiency, environmental toxins, viral infection, changes in Ca^2+^ levels, and oxidative stress, destroy the homeostasis of ER and lead to ER dysfunction [13,14]. Dysfunction in the ER can increase the production of the misfolded or unfolded proteins in ER that can induce ER stress and trigger the unfolded protein response (UPR) [15,16]. The UPR promotes protein folding and post-translational modification by both reducing protein synthesis and increasing the synthesis of molecular chaperones and protein processing enzymes, in order to restore ER homeostasis. Misfolded proteins are degraded by the related proteasomes in ER [17]. There are three signaling pathways of UPR: the inositol-requiring enzyme 1 (IRE1)-mediated pathway; the activated transcription factor 6 (ATF6)-mediated pathway; and the protein kinase R-like endoplasmic reticulum kinase (PERK)-mediated pathway [18]. In the absence of external stimulation, the binding immunoglobulin protein (BIP) inhibits the activation of PERK, IRE1, and ATF6 by binding to them. The external stimulation and the unfolded/misfolded proteins induce the separation of BIP from PERK, IRE1, and ATF6, thereby activating these three molecules. Subsequently, autophosphorylated PERK suppresses mRNA translation and the synthesis of the global protein, and it increases ATF4 expression by phosphorylating eIF2a. Protease 1 (SP1) and protease 2 (SP2) in the Golgi complex then cleaves the isolated ATF6, and the autophosphorylated IRE1 cleaves XBP1 mRNA. Finally, the cleaved XBP1, ATF4, and spliced ATF6 upregulate the expression of the ER chaperone gene and further participate in the elimination of unfolded proteins and the restoration of normal cellular homeostasis (Figure 1) [3,4,19]. When ER stress is at high intensity or lasts for a long time, ER cannot be restored to homeostasis, which induces apoptosis and leads to disease. ER stress induces apoptosis through three pathways, including the caspase-12 kinase pathway, the IRE1/ASK1/JNK pathway, and the C/EBP homologous protein (CHOP)/GADD153 pathway [20]. More and more evidence has shown that ER stress contributes to a variety of diseases, including diabetes, obesity, neurodegenerative diseases, cancer, inflammation, and autoimmune diseases [17,21,22,23,24,25]. It has been reported that the specific inhibitor of PERK promotes glucose-induced insulin secretion and reduces the blood glucose level in a mouse model of type 2 diabetes [26]. The inhibition of IRE1α RNase reduces blood glucose levels and improves beta cell function relative to upregulated serum insulin levels in diabetic mice [27]. This indicates that the inhibition of ER stress can improve the body’s defenses against diabetes. However, the exact mechanism employed is not completely clear.

## 3. Overview of Hydrogen Sulfide

**Figure 2:**
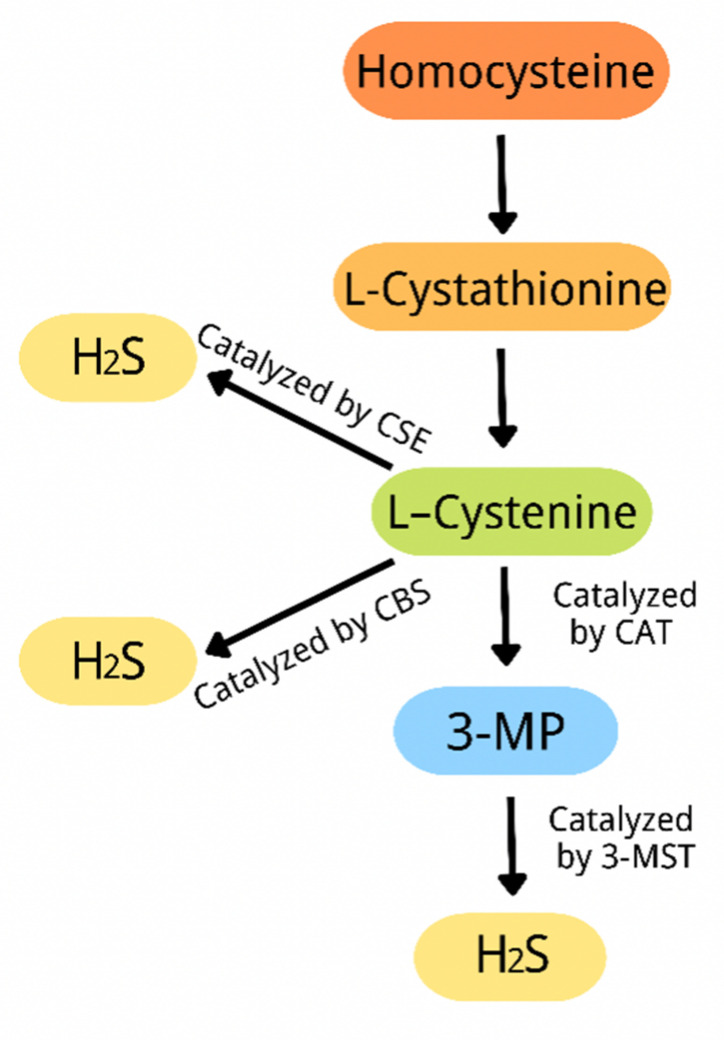
A schematic diagram of the endogenous H_2_S production process.

For a long period of time, hydrogen sulfide (H_2_S) was considered to be merely a toxic and malodorous gas. However, recent studies have revealed that, in addition to its harmful aspects, H_2_S is involved in the regulation of a variety of biological processes. Therefore, it is newly considered to be a gas transmitter after nitric oxide (NO) and carbon monoxide (CO) [28,29,30]. H_2_S in the body is synthesized by three enzymes: cystathionine-γ-lyase (CSE), cystathionine-β-synthase (CBS), and 3-mercaptopyruvate thiotransferase (3-MST) [31]. First, homocysteine is catalyzed by CBS to be converted into L-cystathionine through a β-substitution reaction. Second, L-cystathionine is catalyzed by CSE to produce L-cysteine via the elimination of its α, γ-cysteine. Third, L-cysteine produces H_2_S catalyzed by CSE/CBS through a β elimination reaction. In addition, L-cystine can be converted into 3-mercaptopyruvate (3-MP) catalyzed by cysteine aminotransferase (CAT) by transferring amine to α-ketoglutarate to form 3-mercaptopyruvate (3-MP). Finally, 3-MP can be converted into H_2_S catalyzed by 3-MST (Figure 2) [32,33,34]. Studies have shown that H_2_S is involved in various physiological processes, including inflammatory response, energy metabolism, oxidative stress, mitochondrial biogenesis, and neuroregulation [35,36,37,38]. H_2_S plays the above role mainly by regulating cell function. The mechanisms of H_2_S regulating cell function include the following: DNA methylation, histone modification, transcription factor activity, DNA damage repair, and H_2_S post-translational modification of proteins through s-sulfur hydration [39]. Recent evidence indicates that H_2_S plays a vital role by regulating ER stress in neurological diseases, respiratory diseases, vascular diseases, and endocrine diseases [3]. However, the relevant mechanisms have not yet been fully understood, especially the role H_2_S plays in diabetes by regulating ER stress. Therefore, this review summarized the recent literature on the protective effect of H_2_S against diabetes-related diseases by means of regulating ER stress and carefully analyzed the relevant mechanism, with a view of providing a reference point for future studies.

## 4. Hydrogen Sulfide Improves Diabetes-Associated Cognitive Dysfunction by Regulating Endoplasmic Reticulum Stress

Cognitive dysfunction, a degenerative disease of the central nervous system, is related to neurodegenerative diseases and brain aging. At present, much evidence shows that patients with diabetes can suffer cognitive and memory impairment [40]. The rate of cognitive dysfunction in diabetes patients is 1.5–2 times higher than that in nondiabetic patients [41,42,43]. In recent years, the incidence of diabetes-related cognitive dysfunction has been increasing, becoming a serious social burden [44]. However, because the mechanism of diabetes-associated cognitive dysfunction is not completely clear, there is no effective method for treating this disease [45]. More and more studies indicated that enhancing ER stress contributes to diabetes-induced cognitive dysfunction [21,46,47]. Hence, the inhibition of ER stress can improve diabetes-associated cognitive dysfunction. Wei Zou and colleagues used the intraperitoneal injection of streptozotocin (STZ) to establish a diabetes model in mice. Treatment with NaHS (a H2S-releasing agent) significantly lessened cognitive impairment in diabetic rats. In STZ-induced diabetic rats, ER stress in the hippocampus was significantly increased, as evinced by the increase in glucose-regulated protein 78 (GRP78), cleaved caspase-12, and C/EBP homologous protein (CHOP), all of which were reversed by NaHS, indicating that H_2_S inhibited diabetes-associated ER stress. Additionally, NaHS also noticeably promoted hippocampal endogenous H_2_S production in STZ-induced diabetic rats. Collectively, this evidence suggests that H_2_S ameliorates diabetes-associated cognitive dysfunction most likely by inhibiting hippocampal ER stress [48]. ER stress has been reported to contribute to insulin resistance in diabetes [49], and ER stress-induced apoptosis is associated with neuronal death in many neurodegenerative diseases [50,51]. In the above study, diabetes upregulated ER stress in the rat hippocampus. However, the question of whether the increase in ER stress mediates diabetes-associated cognitive impairment needs to be further addressed by using ER stress inhibitors. In addition, the concentration of H_2_S released by NaHS used in the above study is close to the physiological concentration in vivo, which has no toxic effect on diabetic rats. These levels can promote the production of endogenous H_2_S in the hippocampus of diabetic rats, suggesting that exogenous H_2_S can also improve diabetes-associated cognitive impairment by increasing endogenous H_2_S. Furthermore, previous studies have shown that H_2_S can increase ER stress in beta cells in vitro [52], which contradicts the above conclusion that H_2_S inhibits ER stress in the hippocampus of diabetic rats. The reason for this may be related to different tissue and cell types, a question which needs further research.

In addition to the ER stress mentioned above, synaptic dysfunction is also involved in diabetes-associated cognitive impairment [53,54,55]. The improvement of the plasticity of hippocampal synapses can effectively inhibit the occurrence of cognitive impairment [56,57]. The silencing information regulator 1 (SIRT1) is an NAD^+^-dependent deacetylase that regulates gene expression through histone deacetylation and plays a complex role in a variety of diseases [58,59]. It has been reported that SIRT1 plays a vital role in regulating synaptic plasticity [60,61]. However, its mechanism had not yet been fully understood. Therefore, Juan He et al. conducted a series of experiments and found that NaHS upregulated SIRT1 expression in the hippocampus of diabetic rats induced by STZ. The inhibition of SIRT1 with sirtinol (a special inhibitor of SIRT1) abolished the suppression of NaHS upon diabetes-associated cognitive dysfunction, evidenced by the Y-maze test, the Morris water maze test, and the novel object recognition behavioral test, indicating that SIRT1 mediated H_2_S improvements in diabetes-associated cognitive dysfunction. Additionally, NaHS eliminated diabetes-induced ER stress by decreasing the expressions of GRP78, CHOP, and cleaved caspase-12 in the hippocampus, while sirtinol abrogated the effects of NaHS, indicating that H_2_S inhibited diabetes-induced ER stress by promoting SIRT1. Furthermore, sirtinol also eliminated NaHS-induced increased expressions of hippocampal synapse-related protein (synapsin-1, SYN1) in STZ-induced diabetic rats, indicating that H_2_S improved hippocampal synaptic dysfunction by promoting SIRT1. In sum, H_2_S ameliorated the cognitive dysfunction of STZ-diabetic rats by inhibiting hippocampal ER stress and by improving synaptic dysfunction through upregulating SIRT1 [9]. It can be seen from the above studies that H_2_S improves cognitive dysfunction in diabetic rats by inhibiting hippocampal ER stress through promoting SIRT1. Therefore, the mechanism of how SIRT1 regulates ER needs to be clarified. Based on the studies above, H_2_S regulation of autophagy will become a new strategy to improve diabetes-related cognitive impairment.

## 5. Hydrogen Sulfide Improves Diabetic Cardiomyopathy by Regulating Endoplasmic Reticulum Stress

Diabetes cardiomyopathy (DC) refers to the dysfunction of myocardial function in patients with diabetes, excluding the direct causes of hypertension, coronary artery disease, or valve disease [62,63]. It is characterized by early impaired diastolic function, myocardial hypertrophy, myocardial fibrosis, and systolic dysfunction, and it eventually results in heart failure [64]. DC is currently considered to be a cause of high mortality in patients with diabetes, and there is no effective treatment [65]. More and more studies indicate that ER stress is involved in DC [66,67,68]. Larry A. Barr and colleagues fed rats with a high-fat diet (HFD) to establish a diabetes rat model and then performed a series of experiments. The results showed that HFD reduced the circulating and cardiac H_2_S levels of the diabetic rat models and induced cardiac dysfunction in the diabetic rat model, as evinced by cardiac enlargement, cardiac hypertrophy, and fibrosis, which was restored by exogenous H_2_S [69]. Adiponectin (APN) is a fat factor secreted by the adipose tissue, which contributes to maintaining cardiovascular health [70]. APN has been reported to improve cardiomyopathy [71,72]. Further analysis showed that H_2_S therapy restored HFD-inhibited APN levels and suppressed HG-induced cardiac ER stress. In sum, exogenous H_2_S improved HFD-induced cardiac dysfunction by inhibiting ER stress and restoring HFD-suppressed circulating and cardiac H_2_S [69]. Another study confirmed that the inhibition of ER stress by H_2_S can improve diabetes cardiomyopathy. Rui Yang et al. established a rat model of diabetes by intraperitoneal injection of streptozotocin and subsequently conducted a series of experiments. The results showed that hyperglycemia could significantly reduce cardiac function, significantly damage myocardial ultrastructure, increase the content of MDA in myocardial tissue, reduce the activities of SOD and GSH Px, and increase the mRNA expression of CHOP, GRP78, and caspase-12, while NaHS reversed these changes. These results indicate that exogenous H_2_S improved the DC of diabetic rats, probably by reducing oxidative stress injury and ER stress-induced apoptosis [73]. In the above studies, H_2_S might inhibit ER stress through the following three mechanisms: (1) downregulation of the expression of GRP78 and inhibition of the transcription of CHOP; (2) inhibition of the activation of caspase 12; (3) improvement in the activity of antioxidant enzymes, reduction in the production of ROS and lipid peroxides, and reduction in the accumulation of unfolded or misfolded proteins in the ER. Another study also obtained similar results. Fang Li and colleagues found that H_2_S significantly improved DC by reducing myocardial hypertrophy and myocardial collagen deposition induced by hyperglycemia in STZ-induced diabetic rats. Additionally, H_2_S inhibited ER stress by reducing the expression of GRP78, caspase-12 and CHOP induced by hyperglycemia. Considered collectively, it might be deduced that H_2_S ameliorates DC by suppressing STZ-induced ER stress in diabetic rats [74], a conclusion that needs to be verified by using inhibitors of ER stress. It has been reported that ER stress-induced apoptosis contributes to DC [75]. Furthermore, there are two specific ER-related death pathways related to the apoptosis pathway: The first is the CHOP-dependent pathway [76] and the other is the caspase-12-dependent pathway [77]. Therefore, from the above study, it can be inferred that H_2_S can improve DC by inhibiting ER stress-mediated apoptosis by inhibiting CHOP and caspase-12.

In addition to ER stress, ROS can also induce cardiomyocyte apoptosis [78,79]. Fan Yang et al. found that the sarcoplasmic reticulum of the myocardium in diabetic rats was significantly enlarged, the mitochondria were swollen and deformed, and the cristae were broken, indicating that hyperglycemia led to myocardial tissue damage. Exogenous H_2_S reversed these changes. Similar results were obtained in vitro experiments. The expressions of GRP78, p60 ATF-6, CHOP, and cleaved caspase-12 were all increased in the myocardial tissue of diabetic rats and high glucose (HG)-induced H_9_C_2_ cells, while exogenous H_2_S abolished these changes, suggesting that the hyperglycemia-induced ER stress of cardiomyocytes was inhibited by exogenous H_2_S. Moreover, exogenous H_2_S and NAC (ROS scavenger) notably decreased HG-induced intracellular ROS levels in H_9_C_2_ cells. HG upregulated apoptosis by inducing the nucleus condensation and fragmentation of H_9_C_2_ cells which are characteristic of apoptosis. Exogenous H_2_S and NAC could evidently inhibit apoptosis, indicating that ROS mediated exogenous H_2_S inhibition of HG-induced apoptosis [80]. Mitochondrial fusion protein 2 (Mfn-2), a mitochondrial outer membrane GTPase, plays an important role in regulating mitochondrial fusion and the interaction between ER and mitochondria [81,82]. Abnormal Mfn-2 can lead to cardiovascular disease, metabolic disorders, neurodegenerative disease, and neuromuscular disease [83]. It has been reported that Mfn-2 upregulation promotes cardiomyocyte apoptosis [84]. Exogenous H_2_S reduced the increased expression of Mfn-2 induced by HG in H_9_C_2_ cells. Silencing Mfn-2 with siRNA inhibited HG-induced apoptosis of H_9_C_2_ cells, suggesting that Mfn-2 mediated HG-induced apoptosis of H_9_C_2_ cells. Moreover, Mfn-2 siRNA and H_2_S synergistically inhibited ER stress and the expression of Bax and cyt C induced by HG. Considered collectively, exogenous H_2_S improved myocardial tissue damage by suppressing hyperglycemia-induced ER stress and mitochondrial apoptosis of cardiomyocyte through inhibiting Mfn-2 [80]. The evidence indicates that ROS from mitochondria can initiate ER stress [85,86]. The above study demonstrated that hyperglycemia/HG can upregulate the levels of CHOP, cleaved caspase-12, and ROS, thus promoting ER stress/ROS-induced cardiomyocyte apoptosis. Exogenous H_2_S inhibits cardiomyocyte apoptosis by inhibiting ER stress and ROS production.

Besides ER stress-induced apoptosis, ER stress-induced lipotoxicity also contributes to DC. Runmin Guo and colleagues used STZ/palmitic acid to establish a diabetic mouse model and a diabetes cell model, respectively, and conducted a series of experiments. Their results showed that the level of H_2_S in the serum of patients/rats with DC decreased significantly, and the content of H_2_S and the expression of cystathionine-y-lyase (CSE) in the heart tissue of rats with DC also decreased significantly. The H_2_S levels in the supernatant of AC16 cardiomyocytes treated with PA decreased. Cardiac lipotoxicity is characterized by the increase in TUNEL positive cells and lipid deposition in vivo and in vitro, accompanied by a decrease in the endogenous H_2_S levels. Furthermore, NaHS suppressed ER stress in rats with DC or PA-induced AC16 cells by downregulating the protein expressions of GRP78, CHOP, and caspase-12. The NaHS treatment of AC16 cells could inhibit myocardial injury induced by PA, improve cell viability, and inhibit lipid deposition. Additionally, 4-PBA (an ER stress inhibitor) has similar effects. At the same time, the treatment of diabetic rats with NaHS or 4-PBA reduced cardiac lipotoxicity, as evidenced by the decreased cleaved caspase-3 expression, TUNEL positive cells, and lipid accumulation. Collectively, endogenous H_2_S deficiency is associated with the myocardial injury caused by lipotoxicity. Exogenous H_2_S alleviates PA-induced myocardial injury by suppressing ER stress [87]. It can be seen from the above that ER stress-induced apoptosis mediates the lipotoxicity of DCM. Exogenous H_2_S inhibited the apoptosis and lipotoxicity of DCM by inhibiting ER stress. It has been reported that H_2_S is involved in lipid metabolism [88,89], but its mechanism is far from clear. ER stress also plays a vital role in lipid metabolism [90,91]. However, there are few reports on the involvement of H_2_S in lipid metabolism through ER stress. Therefore, the subject in this regard is worth studying in the future.

Myocardial fibrosis is an important feature of DC. It is caused by the excessive production and deposition of myocardial interstitial collagen, which then it leads to myocardial hypertrophy, myocardial diastolic, and systolic dysfunction, eventually leading to heart failure [92,93]. H_2_S has been reported to improve myocardial fibrosis in diabetes, but its mechanism is not completely clear [94,95]. The results of Maojun Liu et al. showed that, in STZ-treated rats, the arrangement of cardiomyocytes was disordered, and myocardial interstitial fibrosis and type III collagen deposition were obviously enhanced, z was reversed by NaHS, indicating that H_2_S ameliorated myocardial histological changes induced by hyperglycemia. Substantial research revealed that the expression ratio of MMPs/TIMPs in the myocardium of diabetic rats was dysregulated and that the TGF-β1 expression level was downregulated, suggesting that hyperglycemia caused myocardial fibrosis. While H_2_S attenuated hyperglycemia-induced myocardial fibrosis, H_2_S also inhibited cardiomyocyte apoptosis by downregulating caspase-3 level and upregulating Bcl-2 level in diabetic rats [96]. The levels of GSH, SOD, 4-HNE, and MDA, which reflect the oxidative damage in myocardium [97], were decreased by H_2_S, indicating that H_2_S alleviated hyperglycemia-induced myocardial oxidative damage. Moreover, H_2_S inhibited myocardial inflammatory responses in diabetic rats by downregulating TNF-α and NF-κB expressions. The ER stress of the diabetes myocardium was also weakened by H_2_S, evidenced by reductions in eIF2αand GRP94 expression. Meanwhile, the JAK/STAT pathway was also inhibited by H_2_S, as evinced by the decrease in JAK-1/2 and STAT1/3/5/6 expression [96]. The JAK/STAT pathway has been reported to be involved in ER stress [98], and ER stress regulates myocardial fibrosis [25,99]. Furthermore, the evidence indicates that H_2_S mitigates myocardial fibrosis by inhibiting oxidative stress. Therefore, it could be deduced that exogenous H_2_S improved myocardial fibrosis in diabetic rats by inhibiting ER stress by suppressing the JAK/STAT pathway, a hypothesis that needs further confirmation by studies with inhibitors [96]. In the role of H_2_S in improving myocardial fibrosis, the relationship among ER stress, apoptosis, inflammatory response, and oxidative stress needs to be clarified. The regulation of ER stress by H_2_S in cardiomyocytes is expected to be a new strategy for the treatment of myocardial fibrosis in diabetes.

## 6. Conclusions

In recent years, more and more studies show that H_2_S can improve diabetes-related diseases by regulating ER stress. In this review, we summarized research indicating that: (1) exogenous H_2_S ameliorates diabetes-associated cognitive dysfunction, most likely by inhibiting hippocampal ER stress; (2) exogenous H_2_S ameliorates cognitive dysfunction of STZ-diabetic rats by inhibiting hippocampal ER stress and by improving synaptic dysfunction through upregulating SIRT1; (3) exogenous H_2_S improves HFD-induced cardiac dysfunction by inhibiting ER stress and by restoring HFD-suppressed circulating and cardiac H_2_S; (4) exogenous H_2_S improves DC, probably by reducing oxidative stress injury and ER stress-induced apoptosis; (5) exogenous H_2_S improves DC by inhibiting ER stress-mediated apoptosis by inhibiting CHOP and caspase-12; (6) exogenous H_2_S improves myocardial tissue damage by suppressing hyperglycemia-induced ER stress and the mitochondrial apoptosis of cardiomyocyte by inhibiting Mfn-2; (7) exogenous H_2_S alleviates PA-induced myocardial injury by suppressing ER stress; (8) exogenous H_2_S improves myocardial fibrosis in diabetic rats by suppressing ER stress by inhibiting the JAK/STAT pathway, a conclusion that needs further confirmation by testing with the inhibitors (Table 1). As can be seen from the above, exogenous H_2_S plays a protective role against diabetes-related diseases by inhibiting ER stress by upregulating SIRT1 and by inhibiting CHOP and caspase-12, Mfn-2, and the JAK/STAT pathway. Whether H_2_S can inhibit ER stress through other mechanisms to improve diabetes-related diseases is worth studying. As opposed to the demonstrated protective effect of H_2_S on diabetes, some evidence shows that H_2_S induced INS-1E cells apoptosis to aggravate diabetes by promoting ER stress via the activation of the p38 MAPK pathway [52]. The reason for this may be related to the concentration of H_2_S used and different stages of diabetes, a question that needs to be further studied.

The evidence shows that H_2_S regulates ER stress via many signaling pathways, such as the BDNF/TrkB signaling pathway, the Akt-Hsp90 signaling pathway, the NF-κB/MAPK signaling pathway, and the PI3K/Akt signaling pathway [19]. Therefore, the signaling pathways of H_2_S regulating ER stress in diabetes need to be further clarified. In addition, the mechanism of H_2_S regulation of ER stress, especially the questions of under what conditions H_2_S promotes ER stress and under what conditions H_2_S inhibits ER stress, needs further study. Furthermore, ER stress also plays an important role by regulating NLRP3 inflammasomes in diabetes [100]; hence, whether exogenous H_2_S can improve diabetes by regulating ER stress/NLRP3 inflammasomes is a subject worth studying in the future. At present, exogenous H_2_S-releasing agents have many disadvantages and cannot fully meet the research on H_2_S. Therefore, the development of new H_2_S-releasing agents is very important for the clinical application of H_2_S in the future.

It is believed that, with the further development of the relevant research, H_2_S regulation of ER stress will provide a new strategy for the treatment of diabetes.

## Figures and Tables

**Table 1:** The summary of the role of H_2_S regulation of endoplasmic reticulum stress in diabetes-related diseases.

The Type of Diabetes-Related Diseases	The Role H_2_S and Endoplasmic Reticulum (ER) Stress	Experimental Model	References
diabetes-associated cognitive dysfunction	Exogenous H_2_S ameliorates diabetes-associated cognitive dysfunction, most likely through the inhibition of hippocampal ER stress.	Streptozotocin (STZ)-induced diabetic rats	[48]
diabetes-associated cognitive dysfunction	Exogenous H_2_S ameliorates cognitive dysfunction through the inhibition of hippocampal ER stress and improvement of the synaptic dysfunction by upregulating SIRT1.	STZ-induced diabetic rats	[9]
diabetes cardiomyopathy (DC)	Exogenous H_2_S improves high fat diet (HFD)-induced cardiac dysfunction by inhibiting ER stress and by restoring HFD-suppressed circulating and cardiac H_2_S.	HFD-induced diabetic rats	[69]
DC	Exogenous H_2_S improves DC probably through the reduction in oxidative stress injury and ER stress-induced apoptosis.	STZ-induced diabetic rats	[73]
DC	Exogenous H_2_S improves DC through the inhibition of ER stress-mediated apoptosis by suppressing CHOP and caspase-12.	STZ-induced diabetic rats	[74]
DC	Exogenous H_2_S improves myocardial tissue damage by suppressing hyperglycemia-induced ER stress and mitochondrial apoptosis of cardiomyocyte through inhibiting Mfn-2.	STZ-induced diabetic rats	[80]
DC	Exogenous H_2_S alleviates myocardial injury by suppressing ER stress.	STZ/palmitic acid-induced diabetic rat/cardiac cells	[87]
DC	Exogenous H_2_S improved myocardial fibrosis in diabetic rats through suppressing ER stress by inhibiting the JAK/STAT pathway, which needs further confirmation with the inhibitors.	STZ-induced diabetic rats	[96]

## Data Availability

Not applicable.
